# Evaluation of biventricular function in asthmatic children with different severity by new echocardiographic modalities

**DOI:** 10.1186/s12887-025-06028-2

**Published:** 2025-09-09

**Authors:** Rehab Elmeazawy, Osama El Razaky, Doaa El Amrousy, Al Shimaa Badreldeen

**Affiliations:** Department of Pediatrics, Faculty of Medicine, Tanta University, Tanta, Egypt

**Keywords:** Asthma, Children, Echocardiography, Speckle tracking echocardiography

## Abstract

**Background:**

This study aimed to evaluate the impact of asthma severity on biventricular cardiac functions using tissue Doppler imaging (TDI), two-dimensional speckle tracking echocardiography (2D-STE), and three-dimensional speckle tracking echocardiography (3D-STE).

**Methods:**

Sixty-three children with asthma, aged between 5 and 16 years, were enrolled in the study along with 63 matched controls. All participants underwent cardiac assessments, including TDI, 2D-STE, 3D-STE, conventional echocardiography, and pulmonary function testing with spirometry.

**Results:**

Sixty-three asthmatic children with a mean age of 9.96 ± 3 years were enrolled in the patient group. Based on the severity of asthma, they s were categorized into three subgroups: mild, moderate, and severe persistent. The TDI examination revealed a significant decline in right ventricular (RV) diastolic and systolic functions, indicated by lower tricuspid E’/A’ ratio and RV S respectively. Additionally, there was a notable increase in both the RV and LV myocardial performance index (MPI) in the severe asthma group compared to other severity subgroups. Children with severe asthma also demonstrated significantly lower values in three-dimensional global longitudinal strain (3D GLS), three-dimensional global circumferential strain (3D GCS), three-dimensional global area strain (3D GAS), and three-dimensional global radial strain (3D GRS) during (3D-STE examination compared to other severity subgroups. However, there was no discernible difference between the severity subgroups when 2D-STE was used.

**Conclusion:**

TDI and 3D-STE exhibited the ability to identify early biventricular dysfunction in pediatric patients diagnosed with severe bronchial asthma.

## Background

Acute bronchial asthma is one of the most common chronic respiratory diseases in children. It is characterized by chronic inflammation that leads to recurrent episodes of hypoxia and activation of various cytokines, which may result in pulmonary vasoconstriction and ventricular dysfunction [1, 2].

A long-term increased respiratory effort may increase intrathoracic pressure, which in turn may raise the right ventricular afterload and lead to pulmonary hypertension, causing right ventricular hypertrophy and/or dilatation. Moreover, communication between the two ventricles may result in left ventricular dysfunction [3].

Conventional echocardiography has been used for years in the assessment of ventricular function; however, its ability to detect minor changes is limited due to irregular shape and lung hyperinflation. So, this emerges the need for new echocardiographic modalities to early detect subclinical ventricular affection [4].

Tissue Doppler imaging (TDI) is a newer technique that utilizes Doppler principles to assess myocardial systolic and diastolic function by measuring ventricular long-axis motion [5].

Two-dimensional speckle tracking echocardiography (2D-STE) has been extensively documented in pediatric studies due to its ability to identify subclinical myocardial dysfunction in various pathological conditions; however, widespread clinical use continues to be delayed [6]. Three-dimensional STE has emerged as a compelling alternative method that can address certain fundamental limitations associated with 2D-STE. This technique enables rapid and thorough deformation analysis derived from a single acquisition of a complete 3D volume [7].

This study aimed to evaluate the impact of asthma and its severity on biventricular cardiac functions using tissue Doppler imaging (TDI), 2D-STE, and three-dimensional speckle tracking echocardiography (3D-STE).

## Methods

This case-control study was conducted in the Pediatrics Pulmonology Department. The research ethics committee of the Faculty of Medicine approved this research under the number 34,648/4/21. The study was conducted in accordance with the ethical standards of the institutional research committee and the 1964 Helsinki Declaration and its later amendments. Written informed consent was obtained from the patients’ guardians.

The study included 63 children with acute persistent bronchial asthma and the control group included 63 apparently healthy children selected from relatives of our patients and properly matched with the study group in terms of age and sex. The diagnosis and categorization of asthma severity were established in accordance with the guidelines set forth by the 2020 National Asthma Education and Prevention Program (NAEPP) [8]. Subsequently, the 63 patients were subdivided into mild persistent, moderate persistent, and severe persistent asthma.

Patients who experienced an upper or lower airway infection, an asthma flare-up within a month of their cardiac evaluation, children with intermittent asthma, children with thoracic deformities, chronic lung disease, cardiovascular, digestive, rheumatic, osteoarticular, genetic disorders, or any medical condition that affected the cardiac system were all excluded from the study.

All included children underwent a thorough evaluation, including a review of their medical history and a thorough clinical examination. Anthropometric data, including height, weight, and body mass index (BMI), as well as eosinophil count and total immunoglobulin E (Ig E) level, were recorded.

Per consensus standards set by the European Respiratory Society, pulmonary function tests such as the forced vital capacity (FVC), forced expiratory volume in the first second (FEV1), and (FEV1/FVC) ratio were carried out using spirometry (Geratherm Spirostik) on patients and controls.

### Echocardiographic Examination

Echocardiographic examination was performed for all included children with 3.5 MHz, S7, and V3 matrix real-time 3-dimensional probes on Vivid 7 and Vivid 9 (GE Healthcare, Horten, Norway). Digital loops were saved on the echocardiography machine’s hard disc and transferred to a workstation (Echo PAC PC, 113, GE, Horten, Norway) for offline analysis. The American Society of Echocardiography guidelines were followed for conventional echocardiography, TDI, 2D STE, and 3D-STE [9].

#### Conventional Echocardiography

Using M-mode, left ventricular (LV) systolic function was evaluated by measuring LV ejection fraction (LVEF), and left ventricular diastolic function was evaluated using the pulsed wave Doppler to measure mitral E/A ratio, where E (passive LV filling) and A-wave (atrial contraction). The right ventricular systolic function was evaluated by measuring tricuspid annular plane systolic excursion (TAPSE) using the M mode in the apical 4 chamber view and the right ventricular diastolic function was evaluated using the pulsed wave Doppler to measure Tricuspid E/A ratio. the mean pulmonary artery pressure (mPAP) was determined based on the peak pulmonary regurge Doppler signal plus right atrial pressure that was assumed to be 5 mmHg for all participants.

#### Pulsed Wave- Tissue Doppler Imaging (PW TDI)

PW-TDI was performed using an apical four-chamber view. Care was taken to increase the frame rate to be greater than 180 frames/s and to minimize the interrogation angle to the targeted ventricular wall to less than 15°. Tissue Doppler images were obtained from both ventricles at the tricuspid valve (TV) annulus and mitral valve (MV) septal annulus. The S wave represented the systolic function, whereas the E′/A′ ratio represented the diastolic function. The myocardial performance index (MPI) assessed both systolic and diastolic ventricular functions, MPI = (ICT + IRT)/ET, where ICT is the isovolumetric contraction time, IRT is the isovolumetric relaxation time, and ET is the ejection time [10].

#### Speckle-tracking echocardiography (STE)

2D-STE images were obtained at a frame rate of > 40 frames/cycle during three cardiac cycles. An apical chamber image was used to measure LV and RV strain rate (SR). The endocardial border was automatically monitored using the software after modifying the 2D planes. However, if more fine-grained tracking is required, it can be manually modified by the operator.

3D-STE: The area strain was calculated in the longitudinal, circumferential, and radial dimensions using the three-dimensional left ventricular quantification function of Echo Pac. An area of interest (ROI) was automatically created from an endocardial and epicardial mesh as the first step in the analysis of the 3D strain tracking dataset. The operator corrected the shape of the region of interest by using attractor points to pull the nearby region of interest border towards the user’s desired location from the tracking results. From the tracking results, 3D global strain derived several parameters, including longitudinal, circumferential, area, and radial strain. Regional thickening or lengthening indicated positive values while negative values resulted from thinning or shortening.

Echocardiographic examinations were performed by expert pediatric cardiologists with more than 15 years’ experience in the pediatric cardiology field who were blinded to the study groups. Inter-observer variability was assessed in 20 randomly selected patients by repeated analyses of the same cine loop.

### Statistical analysis

The data were loaded into a Microsoft Excel spreadsheet and examined using version 23 of SPSS software. Percentages and numbers were used to describe the data. The normality of the distribution was verified using the Kolmogorov-Smirnov test. Normally distributed quantitative data were described using means ± standard deviation (SD) and were compared using Student’s t-test and ANOVA test, while abnormally distributed quantitative variables were described as median and interquartile range (IQR) and were compared using the Kruskal-Wallis test. Correlations between variables were determined using Pearson’s coefficient. Inter-observer reliability was assessed using intraclass correlation coefficient (ICC). Statistical significance was determined at a P-value of < 0.05.

## Results

### Clinical and Laboratory Data

Sixty-three asthmatic children with a mean age of 9.96 ± 3 years were enrolled in the study. Thirty-six patients were male (57.1%), and fifty-three (84.1%) had a family history of atopic illness. Compared to the control group, asthmatic children had considerably higher weight Z-scores (*p* = 0.045), eosinophilic counts, and total Ig E levels, but significantly lower FEV1 and FEV1/FVC ratios (*P* < 0.001) (Table 1).

**Table 1:** Baseline characteristics of studied groups

	**Bronchial Asthma group** **(n=63)**	**Control group** **(n=63)**	**P value**
Sex, n (%)			
Male	36 (57.1%)	30 (47.6%)	0.448
Female	27 (42.9%)	33 (52.4%)	
Age (year)	9.96±3.00	10.00±2.47	0.957
Height (cm)	135.17 ± 14.27	136.90 ± 14.78	0.636
Weight (Kg)	40.52 ±12.08	36.62± 13.13	0.213
Z score	1.20 ± 1.32	0.55± 1.08	0.045*
BMI (kg/m2)	21.75±3.78	19.98±3.75	0.066
Family history			
Positive	53 (84.1%)	0 (0.0%)	<0.001*
Negative	10 (15.9%)	63 (100.0%)	
Laboratory findings			
Eosinophilia %	2.11±0.94	0.876±1.08	<0.001*
Ig E (IU/ml)	200.0 (80.0-501.0)	40.0 (25.5-80.0)	<0.001*
Pulmonary Function tests			
FVC %	100.98 ± 5.02	100.38 ± 7.10	0.670
FEV1 %	66.32 ± 10.05	96.6 ± 2.55	<0.001*
FEV1/FVC	67.73 ± 10.03	96.67 ± 7.05	<0.001*

*BMI *Body mass index, *Ig E* Immunoglobulin E, *FVC* Forced vital capacity, *FEV1* Forced expiratory volume in the first second, *FEV1 / FVC* Forced expiratory volume in the first second/ forced vital capacity

*P<0.05 significant

The 63 patients were classified into three categories (mild, moderate, and severe) based on the severity of their asthma, with each subgroup comprising 21 individuals. Patients with severe and moderate asthma were older and had higher body weight and height than patients with mild asthma. In addition, children with severe asthma showed significantly lower lung function and higher Ig E levels compared to the other subgroups. Although the difference was not statistically significant, severe asthmatics tended to have higher eosinophil counts (Table 2).

**Table 2:** Baseline characteristics of asthmatic patients according to severity

	**Mild group** **(n=21)**	**Moderate group** **(n=21)**	**Severe group** **(n=21)**	**P value**
Sex, n (%)				
Male	10 (27.8%)	11 (30.6%)	15 (41.7%)	0.256
Female	11 (40.7%)	10 (37.0%)	6 (22.2%)	
Age (year)	8.57±2.86	10.76±2.92	10.55±2.86	0.036*
Height (cm)	128.26± 13.95	138.07±11.73	139.19± 14.94	0.021*
Weight (Kg)	34.95± 10.13	41.381 ± 11.79	45.23± 12.41	0.018*
Z score	1.26± 1.37	0.85 ± 1.23	1.49 ± 1.35	0.293
BMI (kg/m2)	20.82±3.11	21.43±3.9	23.08±4.05	0.129
Laboratory findings				
Eosinophilia	2.02±0.88	2.04±0.78	2.26±1.15	0.675
Ig E (IU/ml)	85.0 (77.5- 180.0)	200.0 (133.5-410.0)	950.0 (210.0-1750.0)	<0.001*
Pulmonary Function tests				
FVC %	100.6± 4.17	99.6 ± 4.00	102.71± 6.27	0.125
FEV1 %	77.9± 3.85	65.76± 2.48	55.29± 4.79	<0.001*
FEV1/FVC	79.71± 4.58	66.0± 2.61	57.42± 4.36	<0.001*
Family history				
Negative	2 (9.5%)	5 (23.8%)	3 (14.3%)	<0.001*
Positive	19 (90.5%)	16 (76.2%)	18 (85.7%)	

*BMI *Body mass index, *Ig E* Immunoglobulin E, *FVC *Forced vital capacity, *FEV1* Forced expiratory volume in the first second, *FEV1 / FVC *Forced expiratory volume in the first second/ forced vital capacity

*P<0.05 significant

### Echocardiographic Examination Results

Asthmatic patients showed significantly lower TAPSE (*p* < 0.001) but significantly higher mPAP when compared to the control group. There was no statistically significant difference between the two groups in terms of LV ejection fraction (LV EF), mitral E/A ratio, and tricuspid E/A ratio. (Table 3)

**Table 3:** Echocardiographic measures among the studied groups

ECHO parameters	Bronchial asthma group (n=63)	Control group(n=63)	P value
Conventional ECHO			
Right ventricle			
-Systolic function			
TAPSE (mm)	18.82±1.26	20.16±1.22	<0.001*
-Diastolic function			
Tricuspid E/A	1.32 ± 0.12	1.40 ± 0.15	0.341
Left ventricle			
-Systolic function			
LV EF %	65.89±3.38	65.00±5.86	0.394
-Diastolic function			
Mitral E/A	1.43±0.17	1.49±0.15	0.142
Right heart Doppler			
mPAP (mmHg)	19.23±5.81	13.29±3.15	<0.001*
TDI parameters			
Right ventricle parameters			
S (cm/s)	4.73±0.84	6.00±0.71	<0.001*
E'/A'	1.16±0.35	1.60±0.46	<0.001*
MPI	0.53±0.07	0.39±0.04	<0.001*
Left ventricle parameters			
S (cm/s)	6.27±1.42	6.71±0.75	0.173
E'/A'	1.50±0.40	1.54±0.21	0.705
MPI	0.53±0.12	0.37±0.07	<0.001*
Speckle Tracking Echocardiography			
LV 2D SR			
LS	-17.06±3.98	-23.05±2.56	<0.001*
RV LS	-18.3 ± 2.4	-24.5 ±3.1	<0.001*
LV 3D SR			
GLS (%)	-15.93±3.82	-21.01±2.30	<0.001*
GCS (%)	-15.00±4.25	-21.62±0.97	<0.001*
GAS (%)	-20.11±3.84	-24.09±2.51	<0.001*
GRS (%)	31.05±7.39	36.95±4.62	<0.001*

*TAPSE* Tricuspid annular plane systolic excursion, *LV EF* Left ventricular ejection fraction, *E/A* Early/Late diastolic velocities, *TDI* Tissue Doppler Imaging, *S* Systolic function, *E'/A*' Early/Late diastolic velocities, *MPI* Myocardial performance index, *2D SR* Two-dimensional strain rate, *LS* Longitudinal strain, *RV LS* Right ventricle longitudinal strain, *mPAP* Mean pulmonary artery pressure, *3D SR* Three-dimensional strain rate, *GLS* Global longitudinal strain, *GCS* Global circumferential strain, *GAS* Global area strain, *GRS G*lobal radial strain

*P<0.05 significant

When comparing the asthmatic group with the control group using TDI, it was found that the RV systolic (S) and diastolic (E’/A’) functions were significantly lower. However, there was no statistically significant difference between the two groups in terms of LV systolic (S) and diastolic (E’/A’) functions. Nevertheless, the myocardial performance index (MPI) of both ventricles was significantly higher (*p* < 0.001) in the asthmatic group than in the healthy group. (Table 3)

Compared to the control group, the mean value of LV two-dimensional longitudinal strain (LV 2D LS) and RV longitudinal strain (RV LS) were significantly lower in patients with asthma (*p* < 0.001). The LV three-dimensional global longitudinal strain (LV 3D-GLS), LV three-dimensional global circumferential strain (LV 3D-GCS), LV three-dimensional global area strain (LV 3D-GAS), and LV three-dimensional global radial strain (LV 3D-GRS) values were significantly lower in the patient group compared to the control group (*P* < 0.001) (Table 3).

The TDI examination results of the severe asthma group showed significantly lower right ventricular diastolic function (E’/A’) and significantly higher MPI when compared to other severity subgroups (*P* = 0.001, *P* = 0.007, respectively). In the TDI examination of the LV, the severe asthma group showed a significantly higher MPI than the other asthma subgroups. Children with severe asthma had significantly lower LV 3D-GLS, LV 3D-GCS, LV 3D-GAS, and LV 3D-GRS during 3D STE examination when compared to other severity subgroups. However, using 2D speckle tracking echocardiography, no difference was observed between the three subgroups as regards LV LS. However, 2D RV LS was significantly lower in patients with severe asthma compared to those with mild or moderate asthma (Table 4).

**Table 4:** Echocardiographic measures of the patient group according to the severity of asthma

ECHO parameters	Mild group (*n* = 21)	Moderate group (*n* = 21)	Severe group (*n* = 21)	*P* value
Conventional ECHO				
Right ventricle				
-Systolic function				
TAPSE (mm)	19.05 ± 1.29	19.10 ± 1.34	18.30 ± 1.12	0.073
-Diastolic function				
Tricuspid E/A	1.36 ± 0.11	1.30 ± 0.09	1.28 ± 0.10	0.0621
Left ventricle				
-Systolic function				
LV EF %	65.81 ± 4.07	65.14 ± 3.20	66.71 ± 2.69	0.323
-Diastolic function				
Mitral E/A	1.44 ± 0.19	1.40 ± 0.18	1.45 ± 0.14	0.550
Right heart Doppler				
mPAP (mmHg)	13.57 ± 3.22	20.24 ± 2.49	24.05 ± 5.39	< 0.001*
TDI parameters				
Right ventricle parameters				
S (cm/s)	4.90 ± 0.70	4.84 ± 0.81	4.44 ± 0.96	0.150
E’/A’	1.32 ± 0.28	1.07 ± 0.36	1.07 ± 0.36	0.001*
MPI	0.49 ± 0.04	0.54 ± 0.06	0.56 ± 0.09	0.007*
Left ventricle parameters				
S (cm/s)	6.62 ± 1.47	6.33 ± 1.02	5.86 ± 1.65	0.215
E’/A’	1.54 ± 0.34	1.51 ± 0.47	1.46 ± 0.41	0.829
MPI	0.46 ± 0.09	0.56 ± 0.11	0.57 ± 0.13	0.003*
Speckle Tracking Echocardiography				
LV 2D SR				
LS	−18.19 ± 3.36	−17.38 ± 3.17	−16.57 ± 4.60	0.384
RV LS	−20.3 ± 3.4	−19.1 ± 2.7	−16.5 ± 2.3	0.01*
LV 3D SR				
GLS (%)	−19.02 ± 1.82	−15.62 ± 2.04	−12.84 ± 4.36	< 0.001*
GCS (%)	−17.24 ± 2.28	−16.95 ± 1.96	−10.37 ± 4.15	< 0.001*
GAS (%)	−21.81 ± 3.59	−20.57 ± 2.44	−17.74 ± 4.33	0.002*
GRS (%)	36.85 ± 4.54	30.48 ± 3.26	25.26 ± 8.50	< 0.001*

*TAPSE* Tricuspid annular plane systolic excursion, *LV EF *Left ventricular ejection fraction, *E/A* Early/Late diastolic velocities, *TDI* Tissue Doppler Imaging, *S* Systolic function, *E’/A*’: Early/Late diastolic velocities, *MPI* Myocardial performance index, *mPAP*: Mean pulmonary artery pressure, *2D SR*: Two-dimensional strain rate, *LS* Longitudinal strain, *RV LS* Right ventricle longitudinal strain, *3D SR*: Three-dimensional strain rate, *GLS* Global longitudinal strain, *GCS* Global circumferential strain, *GAS G*lobal area strain, *GRS* Global radial strain

*P < 0.05 significant

The results of pulmonary function tests (FEV1 and FEV1/FVC ratio) showed a significant negative correlation with weight, BMI, IgE level, mPAP, and MPI, whereas they showed a significant positive correlation with TAPSE, 2D RV LS, LV 3D GLS, LV 3D GCS, LV 3D GAS, and LV 3D GRS (Table 5).

**Table 5:** Correlation between pulmonary function tests (FEV1, FEV1/FVC) and other parameters in the patients group

	FEV1		FEV1/FVC	
	*r*	*p*	*r*	*P*
Age	−0.223	0.079	−0.254	0.045*
Height	−0.212	0.096	−0.241	0.058
Weight	−0.307	0.014*	−0.304	0.015*
Z score	−0.097	0.448	−0.038	0.765
BMI	−0.290	0.021*	−0.251	0.047*
Eosinophilia	−0.125	0.328	−0.101	0.429
Ig E	−0.465	< 0.001*	−0.507	< 0.001*
LV EF	−0.161	0.209	−0.106	0.408
Mitral E/A	−0.065	0.613	−0.048	0.706
TAPSE	0.259	0.041*	0.233	0.066
Tricuspid E/A	−0.122	0.651	−0.324	0.572
MPAP	−0.634	< 0.001*	−0.627	< 0.001*
LV S	0.177	0.164	0.203	0.111
LV E’/A’	0.007	0.954	0.028	0.829
LV MPI	−0.308	0.014*	−0.354	0.004*
RV S	0.072	0.575	0.141	0.269
RV E’/A’	0.255	0.043*	0.303	0.016*
RV MPI	−0.476	< 0.001*	−0.266	0.035*
2D LV LS	0.006	0.966	0.076	0.556
2D RV LS	0.315	0.04*	0.456	0.03*
3D GLS	0.744	< 0.001*	0.699	< 0.001*
3D GCS	0.766	< 0.001*	0.738	< 0.001*
3D GAS	0.624	< 0.001*	0.599	< 0.001*
3D GRS	0.600	< 0.001*	0.544	< 0.001*

r: Pearson coefficient

*BMI* Body mass index,* LV EF *Left ventricular ejection fraction, *E/A* Early/Late diastolic velocities, *TAPSE *Tricuspid annular plane systolic excursion, *mPAP* Mean pulmonary artery pressure, *S* Systolic function, *E’/A’* Early/Late diastolic velocities, *MPI* Myocardial performance index, *RV *Right ventricle, *2D LS* Two dimensional longitudinal strain, *RV LS* Right ventricle longitudinal strain, *3D GLS* Three dimensional global longitudinal strain, *3D GCS* Three dimensional global circumferential strain, *3D GAS *Three dimensional global area strain, *3D GRS* Three dimensional global radial strain

*: Statistically significant at *p* ≤ 0.05

Inter-observer reliability was excellent for 3D-GLS where ICC was (0.89), 3D-GCS where ICC was (0.91), 3D-GCS where ICC was (0.94), and 3D-GRS where ICC was (0.90).

**Fig. 1:**
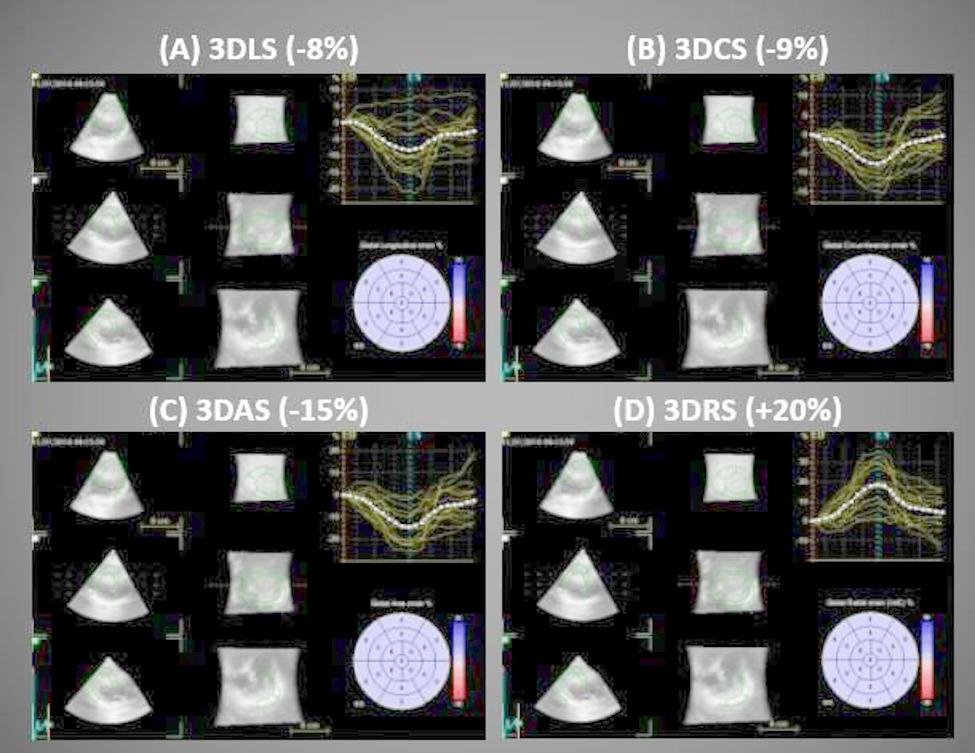
Decreased LV 3D-STE in a patient with severe asthma; A: 3D-GLS, B: 3D-GCS, C: 3D-GAS, D: 3D-GRS

Figure (1) showed decreased LV 3D-STE parameters (3D-GLS, 3D-GCS, 3D-GAS, and 3D-GRS) in patients with severe asthma.

## Discussion

Echocardiography can identify right ventricular systolic and diastolic dysfunction even in patients with mild asthma. However, novel techniques are often needed for the early detection of left ventricular dysfunction in mild to moderate asthma patients [11, 12].

Several studies have evaluated cardiac affection in asthmatic children using conventional and tissue Doppler imaging and 2D-STE. However, to our knowledge, this is the first study to evaluate left ventricular function in asthmatic children using 3D-STE.

Our study indicates that both the systolic and diastolic functions of the right ventricle (RV) appear to be compromised, as evidenced by significant differences in TAPSE measured via M-mode, as well as S, E’/A’, and MPI assessed through tissue Doppler imaging (TDI) between the patient group and the control group. These findings align with those of Shedeed, who identified RV systolic and diastolic dysfunction in children with asthma [11]. The function of inflammatory mediators and their long-term effects on cardiac function, particularly in individuals with severe asthma may help to explain systolic affection in patients with asthma. In the early and late phases of bronchial asthma, a variety of mediators and cytokines are generated, including tumor necrosis factor alpha (TNF α) and interleukins (IL) such IL-1 β, IL-2, IL-6, and IL-8 [11]. Conversely, Abdelmohsen et al. reported that while RV systolic function remained intact, diastolic function was diminished in asthmatic children, attributing this discrepancy to variations in the severity and duration of asthma among the populations they studied [13]. RV diastolic affection can be explained by recurrent hypoxic exposure combined with chronic, prolonged inflammation that causes pulmonary vasoconstriction in asthmatic patients which can progress to pulmonary hypertension with subsequent RV hypertrophy and diastolic dysfunction [11].

The results of our study showed that conventional echocardiography was unable to detect left subclinical ventricular systolic or diastolic dysfunction (LV EF, LV E/A). This limitation arises from the insufficient ability to detect the subtle structural changes in asthma patients, a conclusion confirmed by previous research [3, 14, 15].

Contrary to De-Paula et al., [14] who reported a significant difference between the asthmatic group and the control group regarding E’/A’ ratio, which could be explained by the fact that they used lateral mitral annulus in their group. Measurements showed that left ventricular diastolic parameters (E’/A’ ratio) when assessed by TDI in our study, found no significant differences between the two groups. Consistent with the findings of Ozdemir et al. and Zeybek et al., our results showed that left ventricular systolic function (S) remained unaffected in children with asthma [16, 17].

In agreement with the results of Abdelmohsen et al. and Elseify et al., LV MPI determined by TDI was significantly higher in the asthmatic group than in the control group in our study [13, 18]. In addition, it was higher in patients with severe asthma than in patients with mild and moderate asthma.

The asthmatic group had significantly lower two-dimensional longitudinal strain (2D LS) values. Similar findings were observed by Tuleta et al., [19] whereas Abdelmohsen et al., [13] found no significant differences between the patient and control groups. It should be highlighted that their cohort had normal pulmonary functions and mild to moderate asthma.

The TDI and three-dimensional STE both correlated with FEV1 and FEV1/FVC ratio, suggesting a possible relationship between biventricular and pulmonary functions.

In our study, the patient group had significantly lower 3D GLS, 3D GCS, 3D GAS, and 3D GRS values than the control group. There was no information available comparing the 3D exposure values ​​of asthmatic children.

Although 2D-STE is a useful tool for measuring LV dysfunction, it is constrained by foreshortened views, geometric modeling, and out-of-plane speckle motion. 3D-STE helps to overcome these limitations and enables rapid image acquisition with a shorter scanning time, regardless of operator competence [20].

Inter-observer reliability for 3D-STE parameters reduces observer bias, increases reliability of results, improves clinical confidence, and helps with better decision-making. Furthermore, these new echocardiographic modalities have the potential to assist healthcare professionals in monitoring asthmatic children, enable rapid assessment of cardiac dysfunction, and facilitate the optimization of asthma treatment.

### Study Limitations

The relatively small sample size of our study is one of the limitations, however sample size estimation reported that 55 patients were required to detect a mean difference of −3 in LV GLS with a power of 85%, hence the sample size is considered enough. Another limitation was the unavailability of software for complete RV functional assessment by 3D-STE in our device. Nevertheless, the affection of RV function detected by TDI was considered sufficient to preclude the use of more sophisticated ECHO modalities. Lastly, the effect of asthma medication on cardiac function was not studied.

## Conclusion

Subclinical systolic and diastolic biventricular dysfunction was detected in pediatric patients with severe bronchial asthma using TDI and 3D-STE. The degree of cardiac dysfunction is correlated with the severity of asthma. Therefore, it is recommended that children with severe asthma undergo regular echocardiographic screening using TDI and STE techniques.

## Data Availability

The datasets used and/or analyzed during the current study are available from the corresponding author upon reasonable request.
